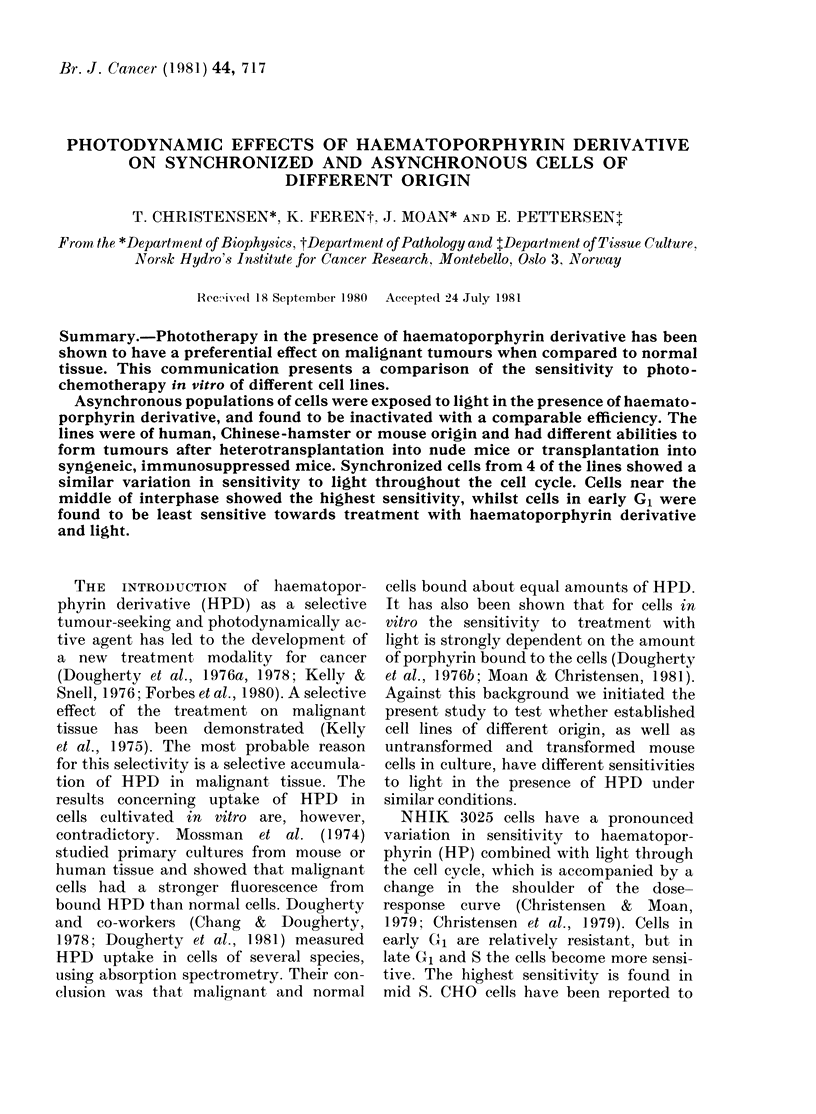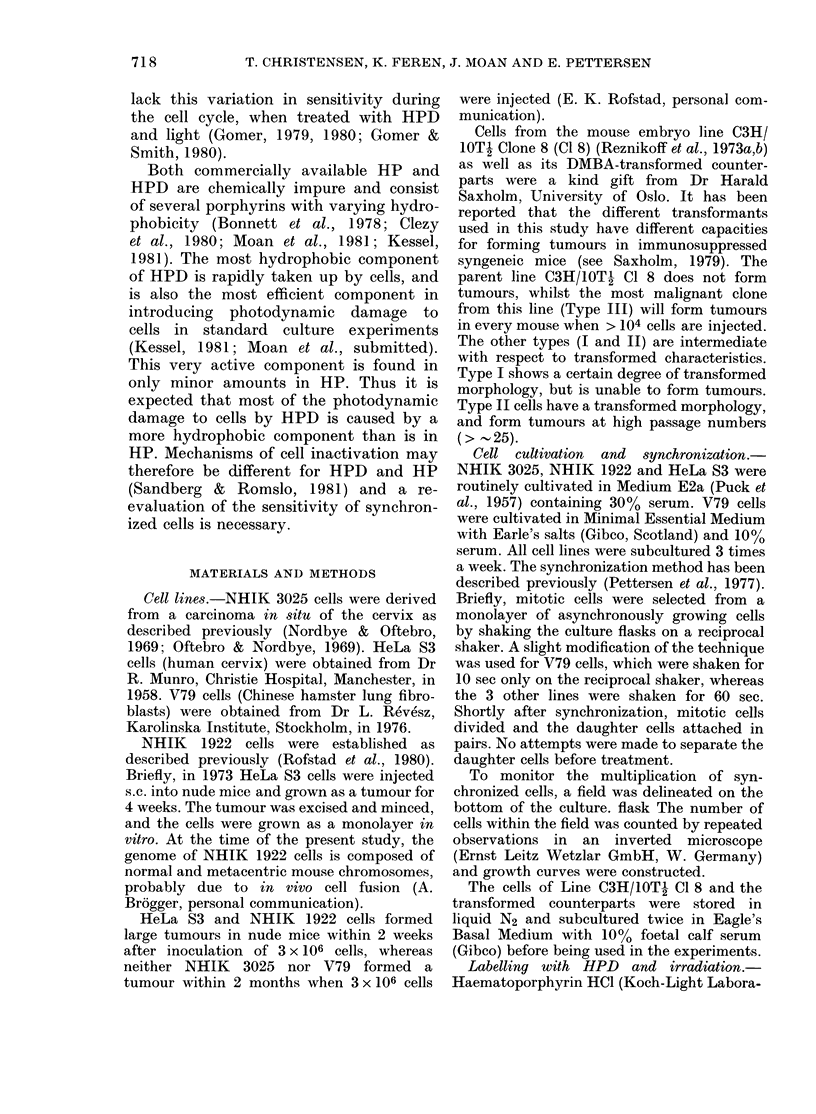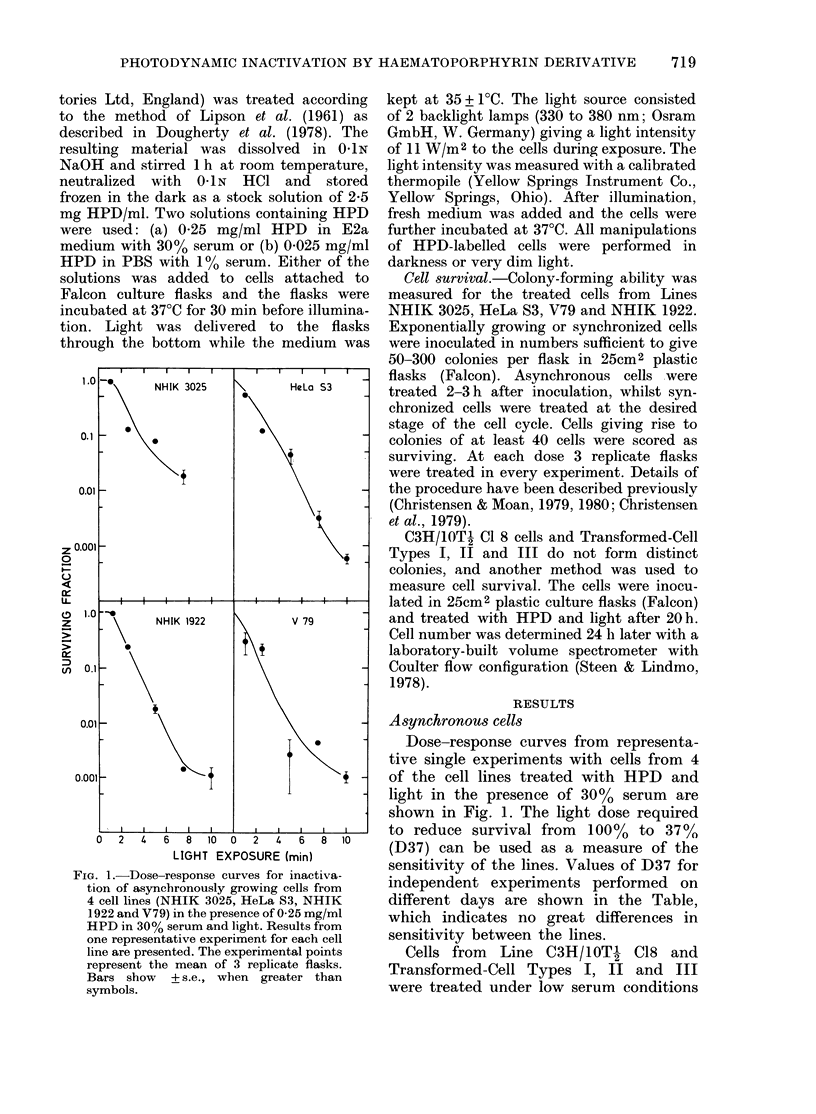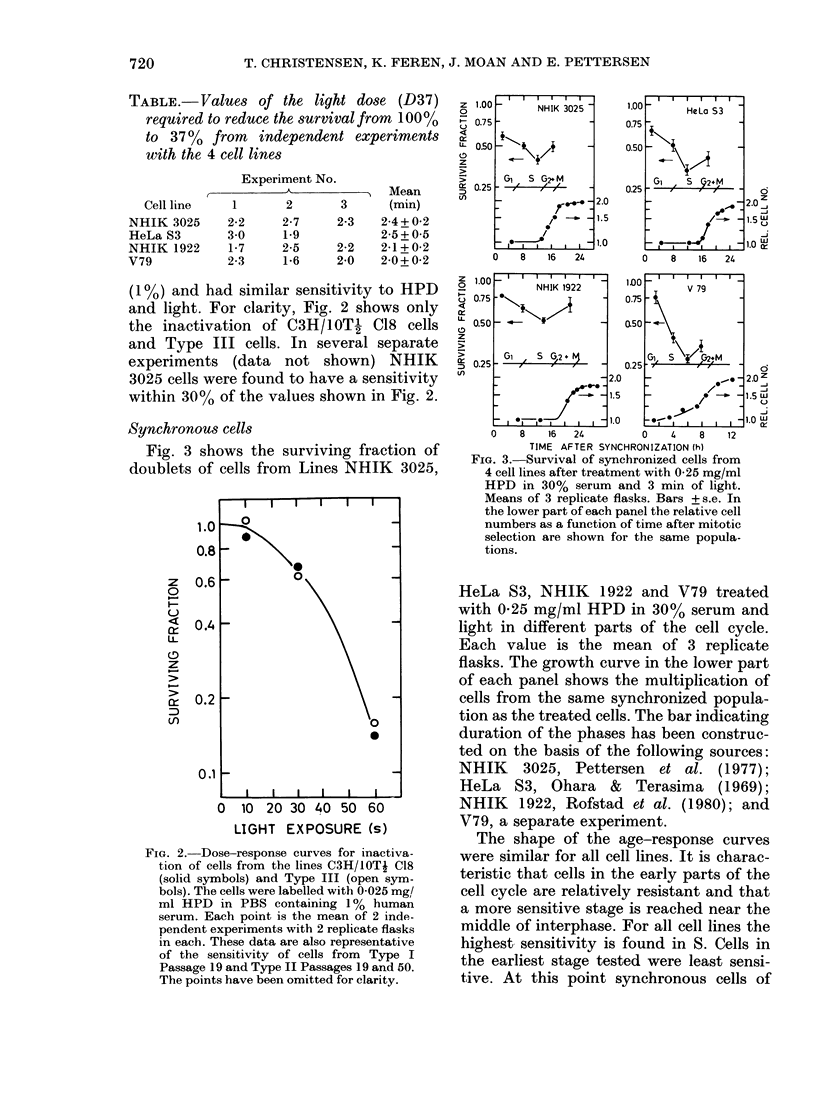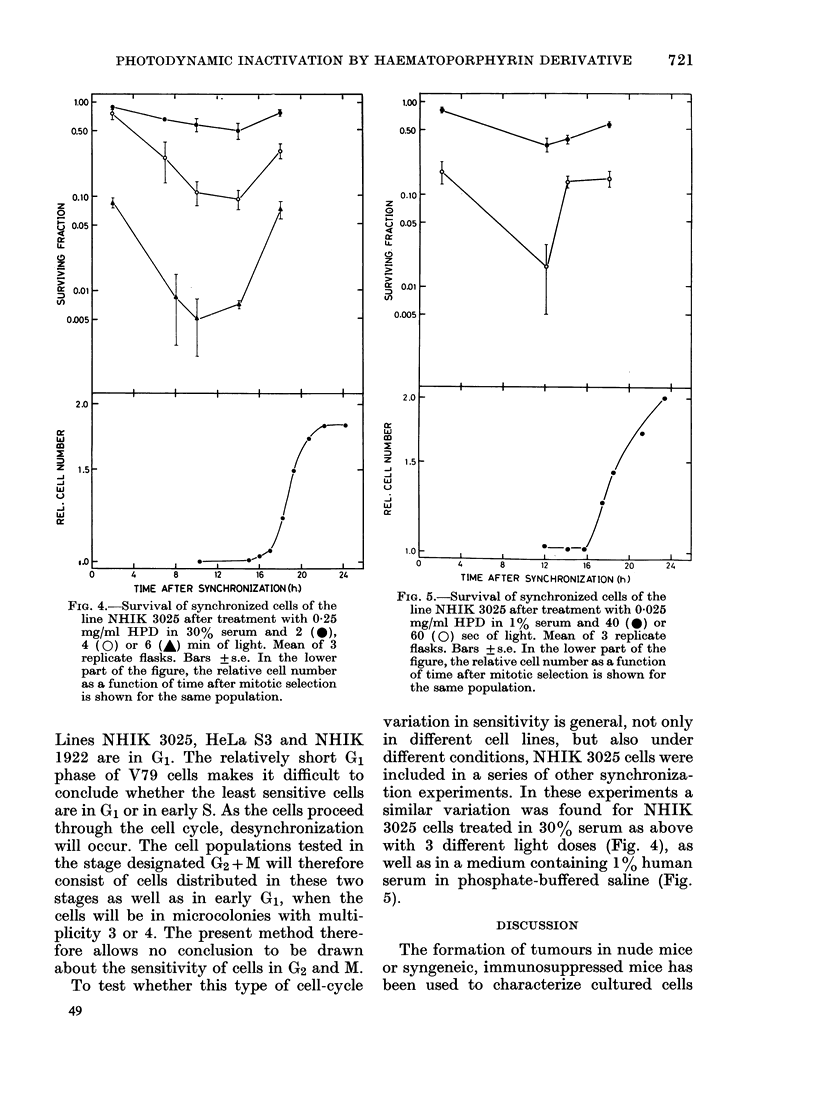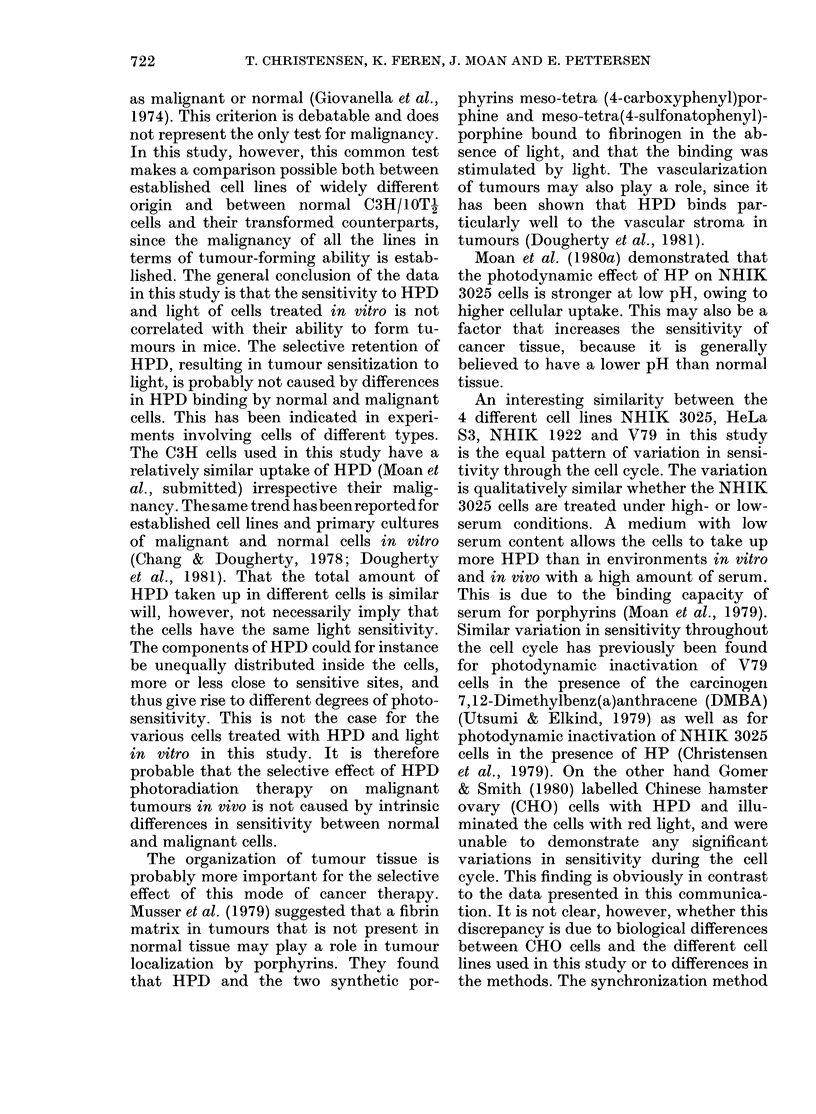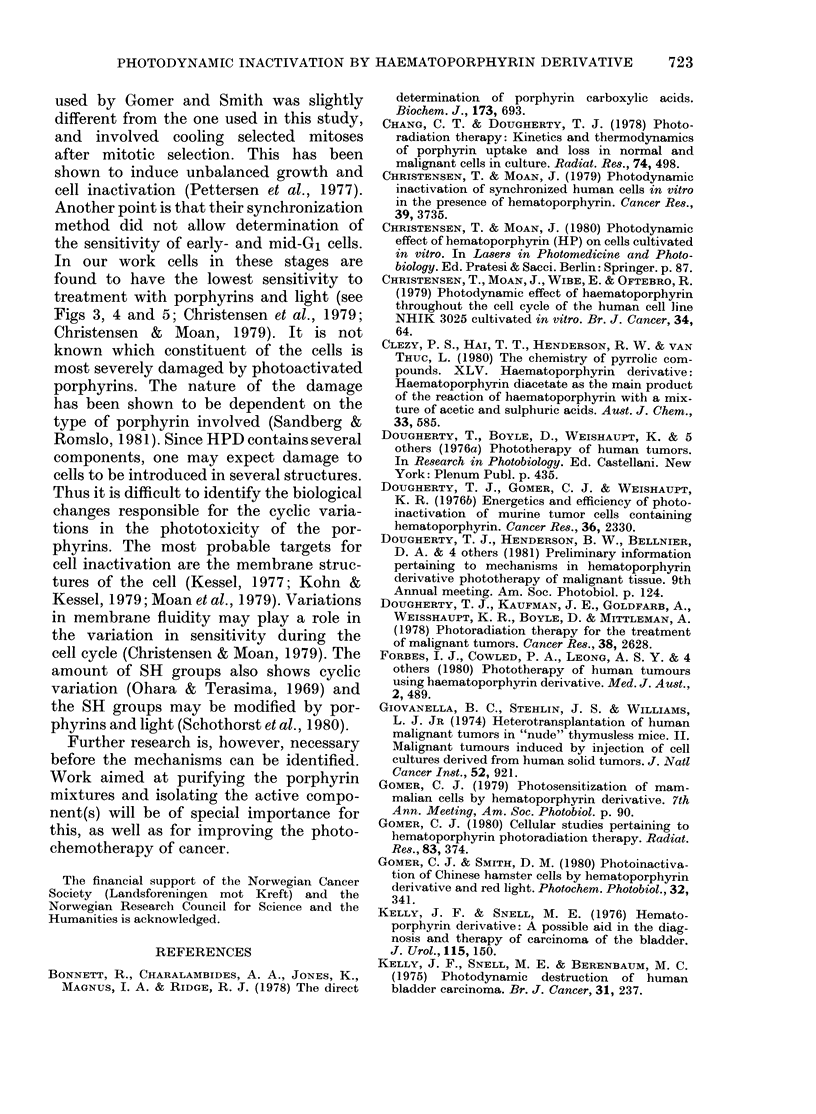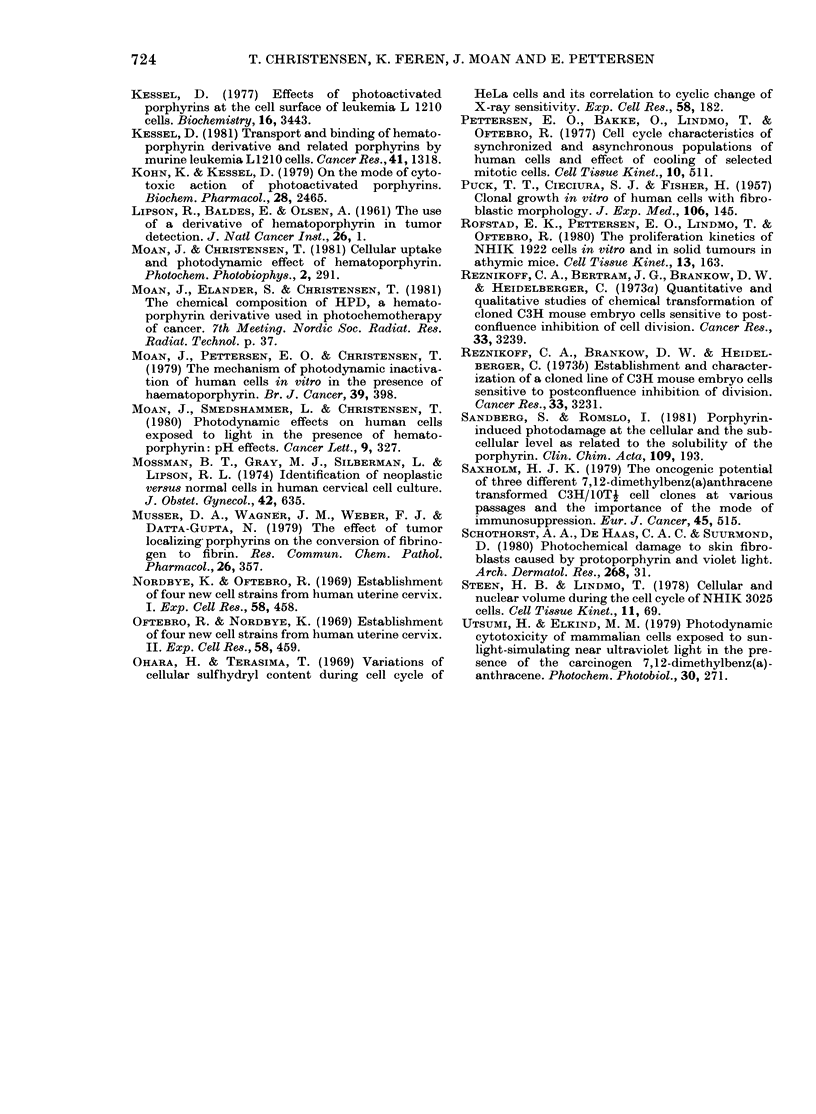# Photodynamic effects of haematoporphyrin derivative on synchronized and asynchronous cells of different origin.

**DOI:** 10.1038/bjc.1981.258

**Published:** 1981-11

**Authors:** T. Christensen, K. Feren, J. Moan, E. Pettersen

## Abstract

Phototherapy in the presence of haematoporphyrin derivative has been shown to have a preferential effect on malignant tumours when compared to normal tissue. This communication presents a comparison of the sensitivity to photochemotherapy in vitro of different cell lines. Asynchronous populations of cells were exposed to light in the presence of haematoporphyrin derivative, and found to be inactivated with a comparable efficiency. The lines were of human, Chinese-hamster or mouse origin and had different abilities to form tumours after heterotransplantation into nude mice or transplantation into syngeneic, immunosuppressed mice. Synchronized cells from 4 of the lines showed a similar variation in sensitivity to light throughout the cell cycle. Cells near the middle of interphase showed the highest sensitivity, whilst cells in early G1 were found to be least sensitive towards treatment with haematoporphyrin derivative and light.


*Br. J. Cancer* (1981) **44**, 717

### PHOTODYNAMIC EFFECTS OF HAEMATOPORPHYRIN DERIVATIVE ON SYNCHRONIZED AND ASYNCHRONOUS CELLS OF DIFFERENT ORIGIN

T. CHRISTENSEN*, K. FEREN†, J. MOAN* AND E. PETTERSEN‡

*From the *Department of Biophysics, †Department of Pathology and ‡Department of Tissue Culture, Norsk Hydro's Institute for Cancer Research, Montebello, Oslo 3, Norway*

Received 18 September 1980 Accepted 24 July 1981

**Summary.—Phototherapy in the presence of haematoporphyrin derivative has been shown to have a preferential effect on malignant tumours when compared to normal tissue. This communication presents a comparison of the sensitivity to photochemotherapy *in vitro* of different cell lines.**

**Asynchronous populations of cells were exposed to light in the presence of haematoporphyrin derivative, and found to be inactivated with a comparable efficiency. The lines were of human, Chinese-hamster or mouse origin and had different abilities to form tumours after heterotransplantation into nude mice or transplantation into syngeneic, immunosuppressed mice. Synchronized cells from 4 of the lines showed a similar variation in sensitivity to light throughout the cell cycle. Cells near the middle of interphase showed the highest sensitivity, whilst cells in early $G_1$ were found to be least sensitive towards treatment with haematoporphyrin derivative and light.**

THE INTRODUCTION of haematoporphyrin derivative (HPD) as a selective tumour-seeking and photodynamically active agent has led to the development of a new treatment modality for cancer (Dougherty *et al.*, 1976*a*, 1978; Kelly & Snell, 1976; Forbes *et al.*, 1980). A selective effect of the treatment on malignant tissue has been demonstrated (Kelly *et al.*, 1975). The most probable reason for this selectivity is a selective accumulation of HPD in malignant tissue. The results concerning uptake of HPD in cells cultivated *in vitro* are, however, contradictory. Mossman *et al.* (1974) studied primary cultures from mouse or human tissue and showed that malignant cells had a stronger fluorescence from bound HPD than normal cells. Dougherty and co-workers (Chang & Dougherty, 1978; Dougherty *et al.*, 1981) measured HPD uptake in cells of several species, using absorption spectrometry. Their conclusion was that malignant and normal cells bound about equal amounts of HPD. It has also been shown that for cells *in vitro* the sensitivity to treatment with light is strongly dependent on the amount of porphyrin bound to the cells (Dougherty *et al.*, 1976*b*; Moan & Christensen, 1981). Against this background we initiated the present study to test whether established cell lines of different origin, as well as untransformed and transformed mouse cells in culture, have different sensitivities to light in the presence of HPD under similar conditions.

NHIK 3025 cells have a pronounced variation in sensitivity to haematoporphyrin (HP) combined with light through the cell cycle, which is accompanied by a change in the shoulder of the dose–response curve (Christensen & Moan, 1979; Christensen *et al.*, 1979). Cells in early $G_1$ are relatively resistant, but in late $G_1$ and S the cells become more sensitive. The highest sensitivity is found in mid S. CHO cells have been reported to lack this variation in sensitivity during the cell cycle, when treated with HPD and light (Gomer, 1979, 1980; Gomer & Smith, 1980).

Both commercially available HP and HPD are chemically impure and consist of several porphyrins with varying hydrophobicity (Bonnett *et al.*, 1978; Clezy *et al.*, 1980; Moan *et al.*, 1981; Kessel, 1981). The most hydrophobic component of HPD is rapidly taken up by cells, and is also the most efficient component in introducing photodynamic damage to cells in standard culture experiments (Kessel, 1981; Moan *et al.*, submitted). This very active component is found in only minor amounts in HP. Thus it is expected that most of the photodynamic damage to cells by HPD is caused by a more hydrophobic component than is in HP. Mechanisms of cell inactivation may therefore be different for HPD and HP (Sandberg & Romslo, 1981) and a re-evaluation of the sensitivity of synchronized cells is necessary.

### MATERIALS AND METHODS

*Cell lines.*—NHIK 3025 cells were derived from a carcinoma *in situ* of the cervix as described previously (Nordbye & Oftebro, 1969; Oftebro & Nordbye, 1969). HeLa S3 cells (human cervix) were obtained from Dr R. Munro, Christie Hospital, Manchester, in 1958. V79 cells (Chinese hamster lung fibroblasts) were obtained from Dr L. Révész, Karolinska Institute, Stockholm, in 1976.

NHIK 1922 cells were established as described previously (Rofstad *et al.*, 1980). Briefly, in 1973 HeLa S3 cells were injected s.c. into nude mice and grown as a tumour for 4 weeks. The tumour was excised and minced, and the cells were grown as a monolayer *in vitro*. At the time of the present study, the genome of NHIK 1922 cells is composed of normal and metacentric mouse chromosomes, probably due to *in vivo* cell fusion (A. Brögger, personal communication).

HeLa S3 and NHIK 1922 cells formed large tumours in nude mice within 2 weeks after inoculation of $3 \times 10^6$ cells, whereas neither NHIK 3025 nor V79 formed a tumour within 2 months when $3 \times 10^6$ cells were injected (E. K. Rofstad, personal communication).

Cells from the mouse embryo line C3H/10T½ Clone 8 (Cl 8) (Reznikoff *et al.*, 1973*a*,*b*) as well as its DMBA-transformed counterparts were a kind gift from Dr Harald Saxholm, University of Oslo. It has been reported that the different transformants used in this study have different capacities for forming tumours in immunosuppressed syngeneic mice (see Saxholm, 1979). The parent line C3H/10T½ Cl 8 does not form tumours, whilst the most malignant clone from this line (Type III) will form tumours in every mouse when $> 10^4$ cells are injected. The other types (I and II) are intermediate with respect to transformed characteristics. Type I shows a certain degree of transformed morphology, but is unable to form tumours. Type II cells have a transformed morphology, and form tumours at high passage numbers ($> \sim 25$).

*Cell cultivation and synchronization.*—NHIK 3025, NHIK 1922 and HeLa S3 were routinely cultivated in Medium E2a (Puck *et al.*, 1957) containing 30% serum. V79 cells were cultivated in Minimal Essential Medium with Earle's salts (Gibco, Scotland) and 10% serum. All cell lines were subcultured 3 times a week. The synchronization method has been described previously (Pettersen *et al.*, 1977). Briefly, mitotic cells were selected from a monolayer of asynchronously growing cells by shaking the culture flasks on a reciprocal shaker. A slight modification of the technique was used for V79 cells, which were shaken for 10 sec only on the reciprocal shaker, whereas the 3 other lines were shaken for 60 sec. Shortly after synchronization, mitotic cells divided and the daughter cells attached in pairs. No attempts were made to separate the daughter cells before treatment.

To monitor the multiplication of synchronized cells, a field was delineated on the bottom of the culture. flask The number of cells within the field was counted by repeated observations in an inverted microscope (Ernst Leitz Wetzlar GmbH, W. Germany) and growth curves were constructed.

The cells of Line C3H/10T½ Cl 8 and the transformed counterparts were stored in liquid $N_2$ and subcultured twice in Eagle's Basal Medium with 10% foetal calf serum (Gibco) before being used in the experiments.

*Labelling with HPD and irradiation.*—Haematoporphyrin HCl (Koch-Light Laboratories Ltd, England) was treated according to the method of Lipson *et al.* (1961) as described in Dougherty *et al.* (1978). The resulting material was dissolved in 0·1N NaOH and stirred 1 h at room temperature, neutralized with 0·1N HCl and stored frozen in the dark as a stock solution of 2·5 mg HPD/ml. Two solutions containing HPD were used: (a) 0·25 mg/ml HPD in E2a medium with 30% serum or (b) 0·025 mg/ml HPD in PBS with 1% serum. Either of the solutions was added to cells attached to Falcon culture flasks and the flasks were incubated at 37°C for 30 min before illumination. Light was delivered to the flasks through the bottom while the medium was

FIG. 1.—Dose–response curves for inactivation of asynchronously growing cells from 4 cell lines (NHIK 3025, HeLa S3, NHIK 1922 and V79) in the presence of 0·25 mg/ml HPD in 30% serum and light. Results from one representative experiment for each cell line are presented. The experimental points represent the mean of 3 replicate flasks. Bars show ±s.e., when greater than symbols.

kept at 35±1°C. The light source consisted of 2 backlight lamps (330 to 380 nm; Osram GmbH, W. Germany) giving a light intensity of 11 W/m$^2$ to the cells during exposure. The light intensity was measured with a calibrated thermopile (Yellow Springs Instrument Co., Yellow Springs, Ohio). After illumination, fresh medium was added and the cells were further incubated at 37°C. All manipulations of HPD-labelled cells were performed in darkness or very dim light.

*Cell survival.*—Colony-forming ability was measured for the treated cells from Lines NHIK 3025, HeLa S3, V79 and NHIK 1922. Exponentially growing or synchronized cells were inoculated in numbers sufficient to give 50–300 colonies per flask in 25cm$^2$ plastic flasks (Falcon). Asynchronous cells were treated 2–3 h after inoculation, whilst synchronized cells were treated at the desired stage of the cell cycle. Cells giving rise to colonies of at least 40 cells were scored as surviving. At each dose 3 replicate flasks were treated in every experiment. Details of the procedure have been described previously (Christensen & Moan, 1979, 1980; Christensen *et al.*, 1979).

C3H/10T½ Cl 8 cells and Transformed-Cell Types I, II and III do not form distinct colonies, and another method was used to measure cell survival. The cells were inoculated in 25cm$^2$ plastic culture flasks (Falcon) and treated with HPD and light after 20 h. Cell number was determined 24 h later with a laboratory-built volume spectrometer with Coulter flow configuration (Steen & Lindmo, 1978).

### RESULTS

### *Asynchronous cells*

Dose–response curves from representative single experiments with cells from 4 of the cell lines treated with HPD and light in the presence of 30% serum are shown in Fig. 1. The light dose required to reduce survival from 100% to 37% (D37) can be used as a measure of the sensitivity of the lines. Values of D37 for independent experiments performed on different days are shown in the Table, which indicates no great differences in sensitivity between the lines.

Cells from Line C3H/10T½ Cl8 and Transformed-Cell Types I, II and III were treated under low serum conditions

TABLE.—*Values of the light dose (D37) required to reduce the survival from 100% to 37% from independent experiments with the 4 cell lines*

| Cell line | Experiment No. 1 | 2 | 3 | Mean (min) |
|---|---|---|---|---|
| NHIK 3025 | 2·2 | 2·7 | 2·3 | 2·4 ± 0·2 |
| HeLa S3 | 3·0 | 1·9 | | 2·5 ± 0·5 |
| NHIK 1922 | 1·7 | 2·5 | 2·2 | 2·1 ± 0·2 |
| V79 | 2·3 | 1·6 | 2·0 | 2·0 ± 0·2 |

(1%) and had similar sensitivity to HPD and light. For clarity, Fig. 2 shows only the inactivation of C3H/10T½ Cl8 cells and Type III cells. In several separate experiments (data not shown) NHIK 3025 cells were found to have a sensitivity within 30% of the values shown in Fig. 2.

*Synchronous cells*

Fig. 3 shows the surviving fraction of doublets of cells from Lines NHIK 3025,

FIG. 2.—Dose-response curves for inactivation of cells from the lines C3H/10T½ Cl8 (solid symbols) and Type III (open symbols). The cells were labelled with 0·025 mg/ml HPD in PBS containing 1% human serum. Each point is the mean of 2 independent experiments with 2 replicate flasks in each. These data are also representative of the sensitivity of cells from Type I Passage 19 and Type II Passages 19 and 50. The points have been omitted for clarity.

FIG. 3.—Survival of synchronized cells from 4 cell lines after treatment with 0·25 mg/ml HPD in 30% serum and 3 min of light. Means of 3 replicate flasks. Bars ± s.e. In the lower part of each panel the relative cell numbers as a function of time after mitotic selection are shown for the same populations.

HeLa S3, NHIK 1922 and V79 treated with 0·25 mg/ml HPD in 30% serum and light in different parts of the cell cycle. Each value is the mean of 3 replicate flasks. The growth curve in the lower part of each panel shows the multiplication of cells from the same synchronized population as the treated cells. The bar indicating duration of the phases has been constructed on the basis of the following sources: NHIK 3025, Pettersen *et al.* (1977); HeLa S3, Ohara & Terasima (1969); NHIK 1922, Rofstad *et al.* (1980); and V79, a separate experiment.

The shape of the age-response curves were similar for all cell lines. It is characteristic that cells in the early parts of the cell cycle are relatively resistant and that a more sensitive stage is reached near the middle of interphase. For all cell lines the highest sensitivity is found in S. Cells in the earliest stage tested were least sensitive. At this point synchronous cells of

FIG. 4.—Survival of synchronized cells of the line NHIK 3025 after treatment with 0·25 mg/ml HPD in 30% serum and 2 (●), 4 (○) or 6 (▲) min of light. Mean of 3 replicate flasks. Bars ±s.e. In the lower part of the figure, the relative cell number as a function of time after mitotic selection is shown for the same population.

FIG. 5.—Survival of synchronized cells of the line NHIK 3025 after treatment with 0·025 mg/ml HPD in 1% serum and 40 (●) or 60 (○) sec of light. Mean of 3 replicate flasks. Bars ±s.e. In the lower part of the figure, the relative cell number as a function of time after mitotic selection is shown for the same population.

Lines NHIK 3025, HeLa S3 and NHIK 1922 are in $G_1$. The relatively short $G_1$ phase of V79 cells makes it difficult to conclude whether the least sensitive cells are in $G_1$ or in early S. As the cells proceed through the cell cycle, desynchronization will occur. The cell populations tested in the stage designated $G_2+M$ will therefore consist of cells distributed in these two stages as well as in early $G_1$, when the cells will be in microcolonies with multiplicity 3 or 4. The present method therefore allows no conclusion to be drawn about the sensitivity of cells in $G_2$ and M.

To test whether this type of cell-cycle variation in sensitivity is general, not only in different cell lines, but also under different conditions, NHIK 3025 cells were included in a series of other synchronization experiments. In these experiments a similar variation was found for NHIK 3025 cells treated in 30% serum as above with 3 different light doses (Fig. 4), as well as in a medium containing 1% human serum in phosphate-buffered saline (Fig. 5).

### DISCUSSION

The formation of tumours in nude mice or syngeneic, immunosuppressed mice has been used to characterize cultured cells as malignant or normal (Giovanella *et al.*, 1974). This criterion is debatable and does not represent the only test for malignancy. In this study, however, this common test makes a comparison possible both between established cell lines of widely different origin and between normal C3H/10T$\frac{1}{2}$ cells and their transformed counterparts, since the malignancy of all the lines in terms of tumour-forming ability is established. The general conclusion of the data in this study is that the sensitivity to HPD and light of cells treated *in vitro* is not correlated with their ability to form tumours in mice. The selective retention of HPD, resulting in tumour sensitization to light, is probably not caused by differences in HPD binding by normal and malignant cells. This has been indicated in experiments involving cells of different types. The C3H cells used in this study have a relatively similar uptake of HPD (Moan *et al.*, submitted) irrespective their malignancy. The same trend has been reported for established cell lines and primary cultures of malignant and normal cells *in vitro* (Chang & Dougherty, 1978; Dougherty *et al.*, 1981). That the total amount of HPD taken up in different cells is similar will, however, not necessarily imply that the cells have the same light sensitivity. The components of HPD could for instance be unequally distributed inside the cells, more or less close to sensitive sites, and thus give rise to different degrees of photosensitivity. This is not the case for the various cells treated with HPD and light *in vitro* in this study. It is therefore probable that the selective effect of HPD photoradiation therapy on malignant tumours *in vivo* is not caused by intrinsic differences in sensitivity between normal and malignant cells.

The organization of tumour tissue is probably more important for the selective effect of this mode of cancer therapy. Musser *et al.* (1979) suggested that a fibrin matrix in tumours that is not present in normal tissue may play a role in tumour localization by porphyrins. They found that HPD and the two synthetic porphyrins meso-tetra (4-carboxyphenyl)porphine and meso-tetra(4-sulfonatophenyl)-porphine bound to fibrinogen in the absence of light, and that the binding was stimulated by light. The vascularization of tumours may also play a role, since it has been shown that HPD binds particularly well to the vascular stroma in tumours (Dougherty *et al.*, 1981).

Moan *et al.* (1980*a*) demonstrated that the photodynamic effect of HP on NHIK 3025 cells is stronger at low pH, owing to higher cellular uptake. This may also be a factor that increases the sensitivity of cancer tissue, because it is generally believed to have a lower pH than normal tissue.

An interesting similarity between the 4 different cell lines NHIK 3025, HeLa S3, NHIK 1922 and V79 in this study is the equal pattern of variation in sensitivity through the cell cycle. The variation is qualitatively similar whether the NHIK 3025 cells are treated under high- or low-serum conditions. A medium with low serum content allows the cells to take up more HPD than in environments *in vitro* and *in vivo* with a high amount of serum. This is due to the binding capacity of serum for porphyrins (Moan *et al.*, 1979). Similar variation in sensitivity throughout the cell cycle has previously been found for photodynamic inactivation of V79 cells in the presence of the carcinogen 7,12-Dimethylbenz(a)anthracene (DMBA) (Utsumi & Elkind, 1979) as well as for photodynamic inactivation of NHIK 3025 cells in the presence of HP (Christensen *et al.*, 1979). On the other hand Gomer & Smith (1980) labelled Chinese hamster ovary (CHO) cells with HPD and illuminated the cells with red light, and were unable to demonstrate any significant variations in sensitivity during the cell cycle. This finding is obviously in contrast to the data presented in this communication. It is not clear, however, whether this discrepancy is due to biological differences between CHO cells and the different cell lines used in this study or to differences in the methods. The synchronization method used by Gomer and Smith was slightly different from the one used in this study, and involved cooling selected mitoses after mitotic selection. This has been shown to induce unbalanced growth and cell inactivation (Pettersen *et al.*, 1977). Another point is that their synchronization method did not allow determination of the sensitivity of early- and mid-$G_1$ cells. In our work cells in these stages are found to have the lowest sensitivity to treatment with porphyrins and light (see Figs 3, 4 and 5; Christensen *et al.*, 1979; Christensen & Moan, 1979). It is not known which constituent of the cells is most severely damaged by photoactivated porphyrins. The nature of the damage has been shown to be dependent on the type of porphyrin involved (Sandberg & Romslo, 1981). Since HPD contains several components, one may expect damage to cells to be introduced in several structures. Thus it is difficult to identify the biological changes responsible for the cyclic variations in the phototoxicity of the porphyrins. The most probable targets for cell inactivation are the membrane structures of the cell (Kessel, 1977; Kohn & Kessel, 1979; Moan *et al.*, 1979). Variations in membrane fluidity may play a role in the variation in sensitivity during the cell cycle (Christensen & Moan, 1979). The amount of SH groups also shows cyclic variation (Ohara & Terasima, 1969) and the SH groups may be modified by porphyrins and light (Schothorst *et al.*, 1980).

Further research is, however, necessary before the mechanisms can be identified. Work aimed at purifying the porphyrin mixtures and isolating the active component(s) will be of special importance for this, as well as for improving the photochemotherapy of cancer.

The financial support of the Norwegian Cancer Society (Landsforeningen mot Kreft) and the Norwegian Research Council for Science and the Humanities is acknowledged.

### REFERENCES

BONNETT, R., CHARALAMBIDES, A. A., JONES, K., MAGNUS, I. A. & RIDGE, R. J. (1978) The direct determination of porphyrin carboxylic acids. *Biochem. J.*, **173**, 693.

CHANG, C. T. & DOUGHERTY, T. J. (1978) Photoradiation therapy: Kinetics and thermodynamics of porphyrin uptake and loss in normal and malignant cells in culture. *Radiat. Res.*, **74**, 498.

CHRISTENSEN, T. & MOAN, J. (1979) Photodynamic inactivation of synchronized human cells *in vitro* in the presence of hematoporphyrin. *Cancer Res.*, **39**, 3735.

CHRISTENSEN, T. & MOAN, J. (1980) Photodynamic effect of hematoporphyrin (HP) on cells cultivated *in vitro*. In *Lasers in Photomedicine and Photobiology*. Ed. Pratesi & Sacci. Berlin: Springer. p. 87.

CHRISTENSEN, T., MOAN, J., WIBE, E. & OFTEBRO, R. (1979) Photodynamic effect of haematoporphyrin throughout the cell cycle of the human cell line NHIK 3025 cultivated *in vitro*. *Br. J. Cancer*, **34**, 64.

CLEZY, P. S., HAI, T. T., HENDERSON, R. W. & VAN THUC, L. (1980) The chemistry of pyrrolic compounds. XLV. Haematoporphyrin derivative: Haematoporphyrin diacetate as the main product of the reaction of haematoporphyrin with a mixture of acetic and sulphuric acids. *Aust. J. Chem.*, **33**, 585.

DOUGHERTY, T., BOYLE, D., WEISHAUPT, K. & 5 others (1976*a*) Phototherapy of human tumors. In *Research in Photobiology*. Ed. Castellani. New York: Plenum Publ. p. 435.

DOUGHERTY, T. J., GOMER, C. J. & WEISHAUPT, K. R. (1976*b*) Energetics and efficiency of photoinactivation of murine tumor cells containing hematoporphyrin. *Cancer Res.*, **36**, 2330.

DOUGHERTY, T. J., HENDERSON, B. W., BELLNIER, D. A. & 4 others (1981) Preliminary information pertaining to mechanisms in hematoporphyrin derivative phototherapy of malignant tissue. 9th Annual meeting. Am. Soc. Photobiol. p. 124.

DOUGHERTY, T. J., KAUFMAN, J. E., GOLDFARB, A., WEISSHAUPT, K. R., BOYLE, D. & MITTLEMAN, A. (1978) Photoradiation therapy for the treatment of malignant tumors. *Cancer Res.*, **38**, 2628.

FORBES, I. J., COWLED, P. A., LEONG, A. S. Y. & 4 others (1980) Phototherapy of human tumours using haematoporphyrin derivative. *Med. J. Aust.*, **2**, 489.

GIOVANELLA, B. C., STEHLIN, J. S. & WILLIAMS, L. J. JR (1974) Heterotransplantation of human malignant tumors in "nude" thymusless mice. II. Malignant tumours induced by injection of cell cultures derived from human solid tumors. *J. Natl Cancer Inst.*, **52**, 921.

GOMER, C. J. (1979) Photosensitization of mammalian cells by hematoporphyrin derivative. *7th Ann. Meeting, Am. Soc. Photobiol.* p. 90.

GOMER, C. J. (1980) Cellular studies pertaining to hematoporphyrin photoradiation therapy. *Radiat. Res.*, **83**, 374.

GOMER, C. J. & SMITH, D. M. (1980) Photoinactivation of Chinese hamster cells by hematoporphyrin derivative and red light. *Photochem. Photobiol.*, **32**, 341.

KELLY, J. F. & SNELL, M. E. (1976) Hematoporphyrin derivative: A possible aid in the diagnosis and therapy of carcinoma of the bladder. *J. Urol.*, **115**, 150.

KELLY, J. F., SNELL, M. E. & BERENBAUM, M. C. (1975) Photodynamic destruction of human bladder carcinoma. *Br. J. Cancer*, **31**, 237.

KESSEL, D. (1977) Effects of photoactivated porphyrins at the cell surface of leukemia L 1210 cells. *Biochemistry*, **16**, 3443.

KESSEL, D. (1981) Transport and binding of hematoporphyrin derivative and related porphyrins by murine leukemia L1210 cells. *Cancer Res.*, **41**, 1318.

KOHN, K. & KESSEL, D. (1979) On the mode of cytotoxic action of photoactivated porphyrins. *Biochem. Pharmacol.*, **28**, 2465.

LIPSON, R., BALDES, E. & OLSEN, A. (1961) The use of a derivative of hematoporphyrin in tumor detection. *J. Natl Cancer Inst.*, **26**, 1.

MOAN, J. & CHRISTENSEN, T. (1981) Cellular uptake and photodynamic effect of hematoporphyrin. *Photochem. Photobiophys.*, **2**, 291.

MOAN, J., ELANDER, S. & CHRISTENSEN, T. (1981) The chemical composition of HPD, a hematoporphyrin derivative used in photochemotherapy of cancer. *7th Meeting. Nordic Soc. Radiat. Res. Radiat. Technol.* p. 37.

MOAN, J., PETTERSEN, E. O. & CHRISTENSEN, T. (1979) The mechanism of photodynamic inactivation of human cells *in vitro* in the presence of haematoporphyrin. *Br. J. Cancer*, **39**, 398.

MOAN, J., SMEDSHAMMER, L. & CHRISTENSEN, T. (1980) Photodynamic effects on human cells exposed to light in the presence of hematoporphyrin: pH effects. *Cancer Lett.*, **9**, 327.

MOSSMAN, B. T., GRAY, M. J., SILBERMAN, L. & LIPSON, R. L. (1974) Identification of neoplastic *versus* normal cells in human cervical cell culture. *J. Obstet. Gynecol.*, **42**, 635.

MUSSER, D. A., WAGNER, J. M., WEBER, F. J. & DATTA-GUPTA, N. (1979) The effect of tumor localizing porphyrins on the conversion of fibrinogen to fibrin. *Res. Commun. Chem. Pathol. Pharmacol.*, **26**, 357.

NORDBYE, K. & OFTEBRO, R. (1969) Establishment of four new cell strains from human uterine cervix. I. *Exp. Cell Res.*, **58**, 458.

OFTEBRO, R. & NORDBYE, K. (1969) Establishment of four new cell strains from human uterine cervix. II. *Exp. Cell Res.*, **58**, 459.

OHARA, H. & TERASIMA, T. (1969) Variations of cellular sulfhydryl content during cell cycle of HeLa cells and its correlation to cyclic change of X-ray sensitivity. *Exp. Cell Res.*, **58**, 182.

PETTERSEN, E. O., BAKKE, O., LINDMO, T. & OFTEBRO, R. (1977) Cell cycle characteristics of synchronized and asynchronous populations of human cells and effect of cooling of selected mitotic cells. *Cell Tissue Kinet.*, **10**, 511.

PUCK, T. T., CIECIURA, S. J. & FISHER, H. (1957) Clonal growth *in vitro* of human cells with fibroblastic morphology. *J. Exp. Med.*, **106**, 145.

ROFSTAD, E. K., PETTERSEN, E. O., LINDMO, T. & OFTEBRO, R. (1980) The proliferation kinetics of NHIK 1922 cells *in vitro* and in solid tumours in athymic mice. *Cell Tissue Kinet.*, **13**, 163.

REZNIKOFF, C. A., BERTRAM, J. G., BRANKOW, D. W. & HEIDELBERGER, C. (1973*a*) Quantitative and qualitative studies of chemical transformation of cloned C3H mouse embryo cells sensitive to postconfluence inhibition of cell division. *Cancer Res.*, **33**, 3239.

REZNIKOFF, C. A., BRANKOW, D. W. & HEIDELBERGER, C. (1973*b*) Establishment and characterization of a cloned line of C3H mouse embryo cells sensitive to postconfluence inhibition of division. *Cancer Res.*, **33**, 3231.

SANDBERG, S. & ROMSLO, I. (1981) Porphyrin-induced photodamage at the cellular and the subcellular level as related to the solubility of the porphyrin. *Clin. Chim. Acta*, **109**, 193.

SAXHOLM, H. J. K. (1979) The oncogenic potential of three different 7,12-dimethylbenz(a)anthracene transformed C3H/10T$\frac{1}{2}$ cell clones at various passages and the importance of the mode of immunosuppression. *Eur. J. Cancer*, **45**, 515.

SCHOTHORST, A. A., DE HAAS, C. A. C. & SUURMOND, D. (1980) Photochemical damage to skin fibroblasts caused by protoporphyrin and violet light. *Arch. Dermatol. Res.*, **268**, 31.

STEEN, H. B. & LINDMO, T. (1978) Cellular and nuclear volume during the cell cycle of NHIK 3025 cells. *Cell Tissue Kinet.*, **11**, 69.

UTSUMI, H. & ELKIND, M. M. (1979) Photodynamic cytotoxicity of mammalian cells exposed to sunlight-simulating near ultraviolet light in the presence of the carcinogen 7,12-dimethylbenz(a)-anthracene. *Photochem. Photobiol.*, **30**, 271.